# MRI staging of haemodynamic congestion and clinical outcomes

**DOI:** 10.1007/s00330-026-12676-4

**Published:** 2026-06-10

**Authors:** Cesare Mantini, Anna Sorella, Daniele Falco, Darien Calvo Garcia, Daniele Petrucci, Mauro Gianni Perrucci, Sabina Gallina, Massimo Caulo, Luca Saba, Alberto Clemente, Mohammed Yunus Khanji, Giovanni Donato Aquaro, Pankaj Garg, Fabrizio Ricci

**Affiliations:** 1 Department of Neuroscience, Imaging and Clinical Sciences, “G.d’Annunzio” University of Chieti-Pescara, Chieti, Italy; 2 ITAB, Institute for Advanced Biomedical Technologies, “G.d’Annunzio” University of Chieti-Pescara, Chieti, Italy; 3 University Radiology Division, Services Department, SS Annunziata Hospital, Chieti, Italy; 4 UdA-TechLab, Research Center, University “G. d’Annunzio” of Chieti-Pescara, Chieti, Italy; 5 Department of Radiology, University of Cagliari, Cagliari, Italy; 6 Multimodal Cardiovascular and Neuroradiological Imaging Unit, Imaging Department, Fondazione Toscana Gabriele Monasterio, Pisa, Italy; 7 Barts Heart Centre, St. Bartholomew’s Hospital, Barts Health NHS Trust, London, UK; 8 Academic Radiology, Department of Surgical, Medical and Molecular Pathology and of Critical Area, University of Pisa, Pisa, Italy; 9 Department of Cardiovascular and Metabolic Health, University of East Anglia, Norwich, UK; 10 University Cardiology Division, Heart Department, SS Annunziata Hospital, ASL 02 Abruzzo, Chieti, Italy

**Keywords:** Haemodynamic congestion, MRI-wedge, Pulmonary blood volume, Myocardial fibrosis, Prognosis

## Abstract

**Objectives:**

To evaluate the prognostic value of cardiovascular magnetic resonance imaging (MRI)–derived left ventricular filling pressure (MRI-wedge) and pulmonary blood volume index (PBVi), and to assess their association with non-invasive markers of myocardial fibrosis.

**Materials and methods:**

MRI-wedge pressure was computed from left-atrial volume and left-ventricular mass, and PBVi was measured from first-pass transit analysis. Patients were assigned to one of four MRI haemodynamic stages based on normal or elevated MRI-wedge and PBVi: stage 1 (normal profile), stage 2 (isolated volume overload), stage 3 (isolated pressure overload), and stage 4 (combined overload). Non-invasive myocardial tissue indices and clinical outcomes were compared across stages. The primary endpoint was a composite of cardiovascular death and cardiac hospitalisation.

**Results:**

Among 262 participants (mean age 52 ± 17 years; 34% women), mean MRI-wedge was 13.4 ± 2.3 mmHg and mean PBVi was 333 ± 150 mL/m². Higher MRI-wedge values were associated with greater PBVi and prolonged pulmonary transit time (both *p* < 0.001) and increased in parallel with native T1 mapping and indexed extracellular volume (iECV, both *p* < 0.001). Over a median follow-up of 30 months, 29 patients (12%) met the primary endpoint. Event-free survival was reduced in patients with MRI-wedge ≥ 15 mmHg, PBVi ≥ 492 mL/m², and iECV ≥ 16 mL/m², and declined progressively across MRI haemodynamic stages, with stage 4 having the lowest survival (*p* < 0.001). In multivariable analysis, the MRI-based haemodynamic congestion staging system (*p* = 0.009) and iECV (HR 1.072; 95% CI 1.003–1.146; *p* = 0.04) each remained independent predictors of adverse events.

**Conclusions:**

MRI-wedge, PBVi and iECV capture complementary and progressive biological features of haemodynamic congestion—from early structural adaptation to overt circulatory overload–and identify patients at increased risk of adverse clinical events.

**Key Points:**

***Question*** *How do MRI-derived markers of haemodynamic congestion relate to one another, and can their integration improve non-invasive prognostic stratification*?

***Findings*** *Higher MRI-wedge was associated with increased PBVi, prolonged pulmonary transit time, and extracellular matrix expansion. A four-stage haemodynamic congestion grading framework predicted clinical outcomes*.

***Clinical relevance*** *An integrated MRI-based system combining filling-pressure surrogates and pulmonary blood volume identifies progressively higher haemodynamic congestion states, while iECV provides complementary tissue-level prognostic information*.

**Graphical Abstract:**

**Figure:**
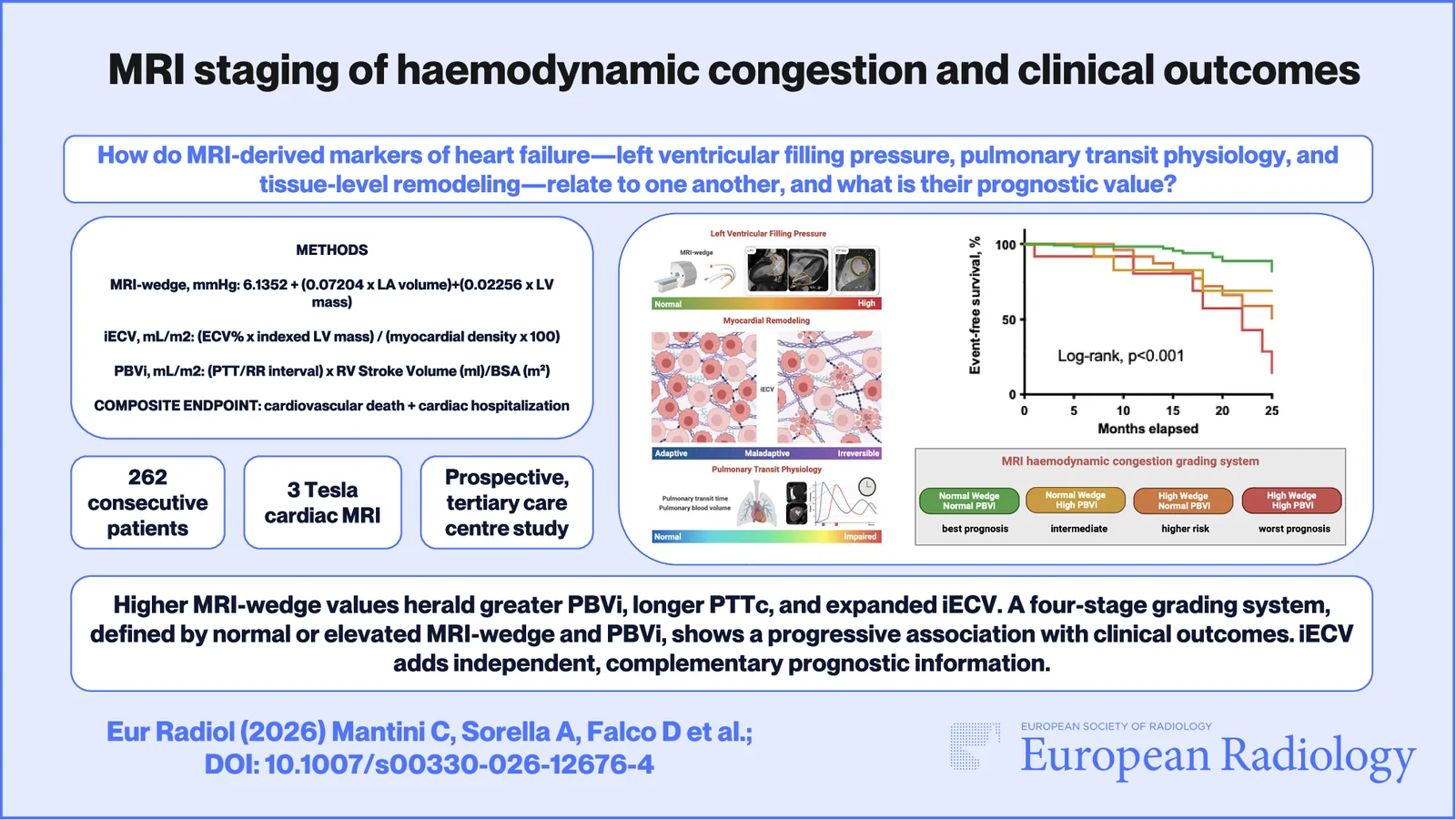

## Introduction

Heart failure (HF) affects more than 25 million individuals worldwide and remains strongly driven by the growing prevalence of obesity and diabetes [1]. It represents the final common pathway for a wide range of cardiovascular disorders, including hypertension, coronary artery disease, valvular abnormalities, genetic cardiomyopathies and congenital disorders. Identifying haemodynamic congestion and quantifying its severity are central diagnostic and therapeutic priorities, as elevated left ventricular filling pressure (LVFP) is a defining physiological feature with major prognostic implications [2].

Right heart catheterisation (RHC) is the reference method for assessing congestion through direct measurement of right atrial pressure and pulmonary artery wedge pressure (PAWP) [3]. When left atrial and pulmonary venous anatomy are preserved, PAWP reliably reflects LVFP [4]. However, the invasive nature and limited availability of RHC restrict its application in routine clinical assessment.

Magnetic resonance imaging (MRI) provides a non-invasive alternative capable of characterising structure, function, and tissue composition while also capturing central circulatory dynamics. Pulmonary blood volume (PBV), derived as the product of pulmonary transit time (PTT) and cardiac output, reflects the effects of increased LVFP on central volume distribution [5, 6]. PTT-derived indices have shown prognostic value in patients with HF [6–11] and in those with pulmonary arterial hypertension [12]. More recently, a simple MRI-based physiological model integrating left atrial volume (LAV) and left ventricular mass (LVM) has demonstrated accuracy in estimating PAWP, outperforming echocardiography and providing prognostic information comparable to invasive RHC [13, 14].

In this study, we examined the association of MRI-wedge pressure and pulmonary transit measures with myocardial fibrosis and outcomes both in individuals with structurally normal heart and in patients with cardiac disease.

## Materials and methods

### Study design and population

This prospective, single-centre study enrolled consecutive patients who underwent cardiovascular MRI at our institution between January and June 2021. All participants provided written informed consent. Exclusion criteria were contraindications to MRI or gadolinium-based contrast administration, and the presence of atrial fibrillation or frequent ventricular ectopy not responsive to antiarrhythmic therapy during acquisition. The final imaging cohort, therefore, comprised 262 outpatients. The study protocol was approved by the Institutional Ethics Committee of Chieti-Pescara and complied with the principles of the Declaration of Helsinki.

### Cardiovascular magnetic resonance

All examinations were performed on a 3.0-Tesla scanner (Achieva D-Stream; Philips Medical Systems) using an eight-channel phased-array cardiac synergy coil. Cine imaging was acquired using balanced steady-state free precession sequences after three-plane scout localisation. Short-axis stacks (8–14 contiguous slices; slice thickness 8 mm; no gap) and standard 2-, 3-, and 4-chamber views were obtained (repetition/echo times 2 ms; flip angle 45°; field of view 40 cm; 30 cardiac phases; matrix 256 × 256; 12–20 views per segment). Quantitative ventricular assessment used MR WorkSpace version 2.6.3.5 (Philips). Pulmonary artery flow was measured with breath-hold, velocity-encoded phase-contrast (PC) cine imaging (TR 4.60–4.92 ms; TE 2.76–3.05 ms; flip angle 15°; 24 phases; pixel spacing 1.32–2.07 mm; slice thickness 10 mm; matrix 256×208; Velocity encoding 150 cm/s, adjusted if aliasing occurred). First-pass perfusion imaging was performed following bolus administration of gadolinium contrast (gadoterate meglumine, Dotarem®, Guerbet – 0.05 mmol/kg at an infusion rate of 4 mL/s), followed by saline flush (4 mL/s). Three short-axis slices (basal, mid-ventricular, apical) were obtained with a field of view of 30 cm; slice thickness 10 mm; repetition/echo times 6/1.1 ms; flip angle 20°; bandwidth 25 Hz; 60 phases; acquisition matrix 128 × 128; reconstruction matrix 256 × 256. Acquisition began concurrently with contrast injection.

### Image analysis

All datasets were analysed offline by two experienced operators (C.M. and F.R.), blinded to clinical and follow-up data, using MR WorkSpace 2.6.3.2 and Segment (Medviso AB, version 4.1.0.1). Biventricular volumes, ejection fraction, and left-ventricular mass were measured on cine short-axis images. Left and right atrial areas were quantified at end-systole in the four-chamber view, excluding atrial appendages and pulmonary veins. Pulmonary artery and aortic flows were calculated from PC images.

### Peak atrial longitudinal strain analysis

Left atrial longitudinal strain was quantified using the strain module of the Segment software (Medviso AB, version 4.1.0.1). Endocardial borders were manually drawn at end-diastole in the four-chamber cine view, and tracking was automatically propagated throughout the cardiac cycle. Tracking quality was visually inspected, with manual adjustments when necessary. Peak atrial longitudinal strain (PALS) was defined as the peak positive strain during the reservoir phase.

### PAWP estimation

**Fig. 1:**
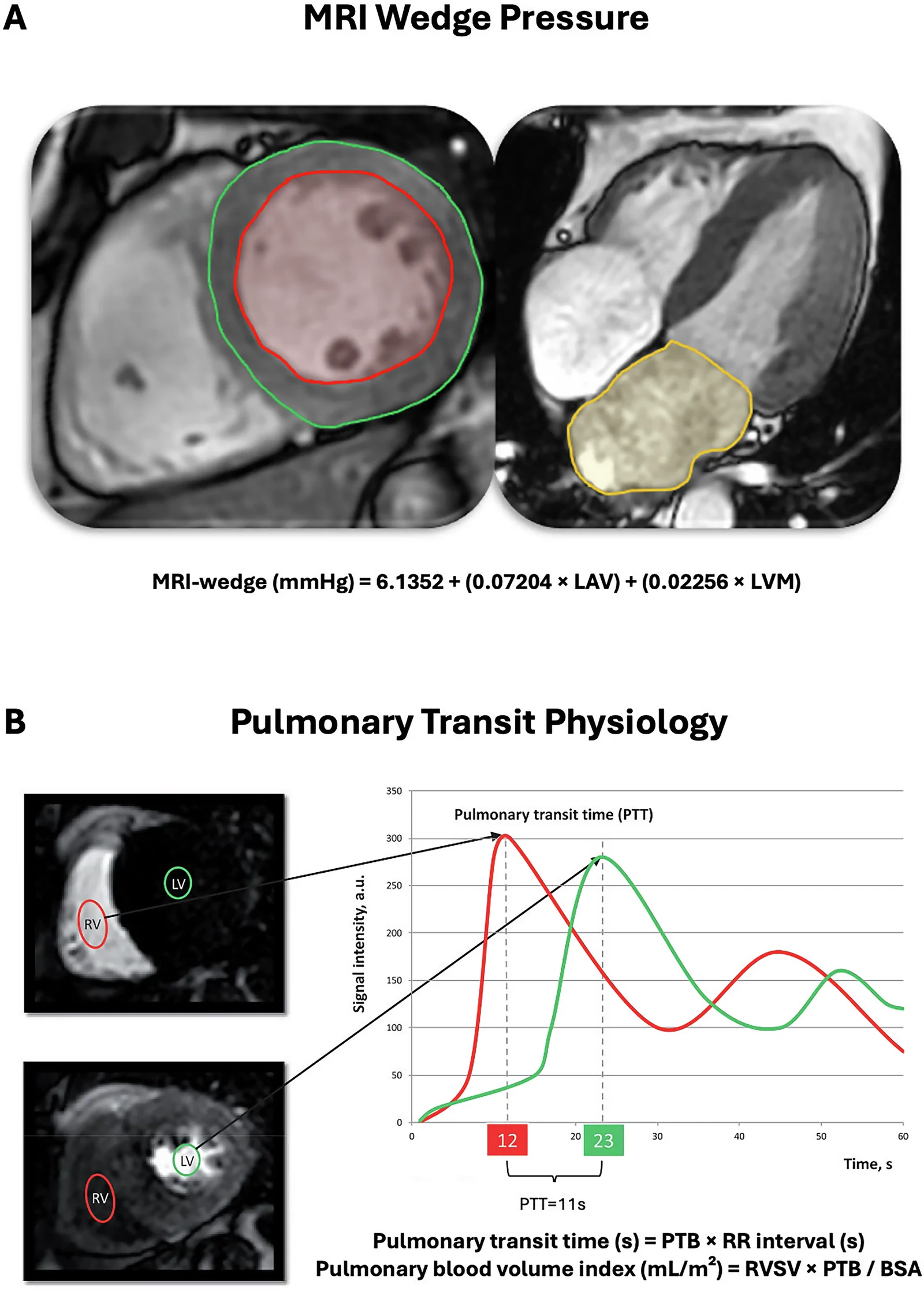
MRI-based quantification of haemodynamic congestion. **A** Estimation of left-ventricular filling pressure from cardiovascular MRI. LAV is measured from long-axis cine images and left-ventricular mass (LVM) from short-axis cine stacks. These parameters are combined in a validated model to estimate pulmonary capillary wedge pressure (MRI-wedge), a non-invasive surrogate of left-ventricular filling pressure. **B** Assessment of pulmonary transit physiology using first-pass contrast MRI. Regions of interest are placed in the right-ventricular (RV) and left-ventricular (LV) cavities to generate signal-intensity curves. Pulmonary transit time (PTT) corresponds to the peak-to-peak interval between RV and LV enhancement. Pulmonary blood volume indexed to body surface area (PBVi) is derived from pulmonary transit beats (PTB) and right-ventricular stroke volume (RVSV)

Non-invasive estimation of LVFP by MRI was performed using the method proposed by Garg et al [13], employing the following equation: MRI-wedge = 6.1352 + (0.07204 × LAV) + (0.02256 × LVM), where LAV represents left atrial volume, and LVM is left ventricular mass (Fig. 1A).

### Pulmonary circulation metrics

Pulmonary transit time (PTT) was measured as the time interval required for a bolus of contrast agent to pass from the right ventricle to LV. During first-pass perfusion imaging, one image per heartbeat was acquired over 60 beats. Regions of interest were placed in the right-ventricular and left-ventricular cavities to generate signal-intensity/beat curves. PTT was defined as the peak-to-peak interval between the two curves. Bazett’s formula was used to correct the PTT interval for the heart rate: Corrected pulmonary transit time (PTTc)(s) = PTT(s)/√R-R interval(s). Pulmonary transit beats (PTB) represented the corresponding number of cardiac cycles. Peak-to-peak measurement was selected due to its high reproducibility (intraclass correlation coefficients 0.99 intra-observer and 0.97 inter-observer) as reported previously [8, 15]. PBV was calculated as the product of PTB and anterograde right ventricular stroke volume (RVSV), indexed to body surface area as PBVi (mL/m²) (Fig. 1B).

### Parametric mapping

Native and post-contrast T1 mapping and T2 mapping were performed using MOLLI and GraSE sequences. ECV was calculated from myocardial and blood T1 values corrected for haematocrit. Indexed extracellular myocardial volume (iECV) was obtained by multiplying ECV by indexed left-ventricular myocardial volume, as previously reported [16].

### MRI haemodynamic congestion staging system

Patients were classified into a four-stage grading system based on the combined status of MRI-derived wedge pressure and PBVi. Elevated LVFP was defined as MRI-wedge ≥ 15 mmHg, consistent with contemporary non-invasive and invasive haemodynamic criteria. Increased central blood volume was defined as PBVi ≥492 mL/m², a threshold derived from prior validation work [8]. Using these cut-offs, four mutually exclusive stages were defined:

- stage 1 (normal profile): normal MRI-wedge and normal PBVi;
- stage 2 (isolated volume overload): normal MRI-wedge with elevated PBVi;
- stage 3 (isolated pressure overload): elevated MRI-wedge with normal PBVi;
- stage 4 (combined overload): both elevated MRI-wedge and elevated PBVi.

This approach allowed integration of upstream LVFP and downstream pulmonary circulatory burden into a single categorical variable, reflecting complementary components of haemodynamic congestion. The model was pre-specified to minimise collinearity in multivariable analyses by replacing individual continuous measures (MRI-wedge and PBVi) with their combined categorical representation. The staging system was used for descriptive comparisons, Kaplan–Meier analysis, and multivariable Cox regression.

### Clinical follow-Up

Clinical follow-up was extended until November 2023 and was conducted by telephone contact. Causes of death were classified using the modified Hinkle–Thaler system. Non-cardiac deaths were censored at the time of the event.

The primary endpoint was a composite of cardiovascular mortality and cardiac hospitalisation, including presentations for HF or device implantation.

### Statistical analysis

Continuous variables were summarised as mean ± standard deviation or as median with interquartile range, according to distribution. Categorical variables were reported as counts and percentages. Group comparisons used Student’s *t*-test or one-way analysis of variance (ANOVA) for continuous variables and the χ² test for categorical variables. Associations between continuous variables were examined using Pearson correlation coefficients. For variables expected to vary across ordered strata, ANOVA with post-hoc testing was applied.

Time-to-event analyses were performed using Kaplan–Meier estimates, with between-group differences assessed by the log-rank test. Survival curves were stratified according to guideline-based thresholds for elevated LVFP (MRI-wedge ≥ 15 mmHg), the PBVi threshold of 492 mL/m², and the cohort-specific median value for iECV, used in the absence of population reference ranges.

The primary endpoint was evaluated using Cox proportional-hazards models. Univariate models were first generated for all candidate predictors. Variables with biological plausibility and significant univariate association were considered for multivariable analysis. Because MRI-wedge and PBVi represent interdependent components of circulatory congestion, multivariable modelling incorporated the four-stage MRI haemodynamic congestion system as a single categorical variable. iECV, reflecting myocardial tissue remodelling, was evaluated separately as an independent covariate. Hazard ratios with 95% confidence intervals were reported. The proportional-hazards assumption was assessed using Schoenfeld residuals and inspection of residual plots, with no violations detected. All tests were two-sided, with a significance threshold of *p* < 0.05. Analyses were performed using Wizard 2 for Mac (version 2.0.20).

## Results

Of 272 patients screened, 10 were excluded prior to imaging analysis due to atrial fibrillation (*n* = 7) and frequent ventricular ectopy not responsive to antiarrhythmic therapy (*n* = 3). A total of 262 patients (mean age 52 ± 17 years; 34% female) were included in the final imaging cohort. Of these, 54 patients (20.6%) had no structural heart disease, 104 (39.7%) had non-ischaemic cardiomyopathy, 44 (16.8%) had ischaemic heart disease, 4 (1.5%) had valvular heart disease, 7 (2.7%) had congenital heart disease, and 49 (18.7%) presented with other conditions, including myocarditis (*n* = 41), pericarditis (*n* = 2), and cardiac masses (*n* = 6). In patients with cardiac masses, lesions were non-obstructive, did not produce inflow or outflow gradients, and were not considered haemodynamically significant at the time of imaging. Baseline imaging and clinical characteristics are reported in Table 1.

**Table 1:** Baseline characteristics of the study cohort

Covariate	*n* = 262
Age, years	52 ± 17
Female Sex, %	34
LVEDVI, mL/m^2^	81 ± 31
LVEF, %	55 ± 14
LVMI, g/m^2^	68 ± 18
LVMWT, mm	10.5 ± 3
RVEDVI, mL/m^2^	73 ± 24
RVEF, %	59 ± 10
LGE binary, %	76
Native T1 mapping, ms	1266 ± 64
ECV, %	26 ± 5
iECV, mL/m^2^	17 ± 7
T2 mapping, ms	51 ± 9
LA area index, cm^2^/m^2^	23 ± 6
RA area index, cm^2^/m^2^	11 ± 3
LAV, mL	60 ± 27
LAV index, mL/m^2^	33 ± 15
LA ejection fraction, %	28 ± 17
PALS, %	14 ± 9
PTB	8 ± 4
PTT, s	7.9 ± 3
PTTc, s	8 ± 3
PBV, mL	624 ± 284
PBVi, mL/m^2^	333 ± 150
MRI-wedge, mmHg	13.4 ± 2.3

Data presented as mean ± standard deviation unless otherwise indicated

### MRI biomarkers across diagnostic categories

**Fig. 2:**
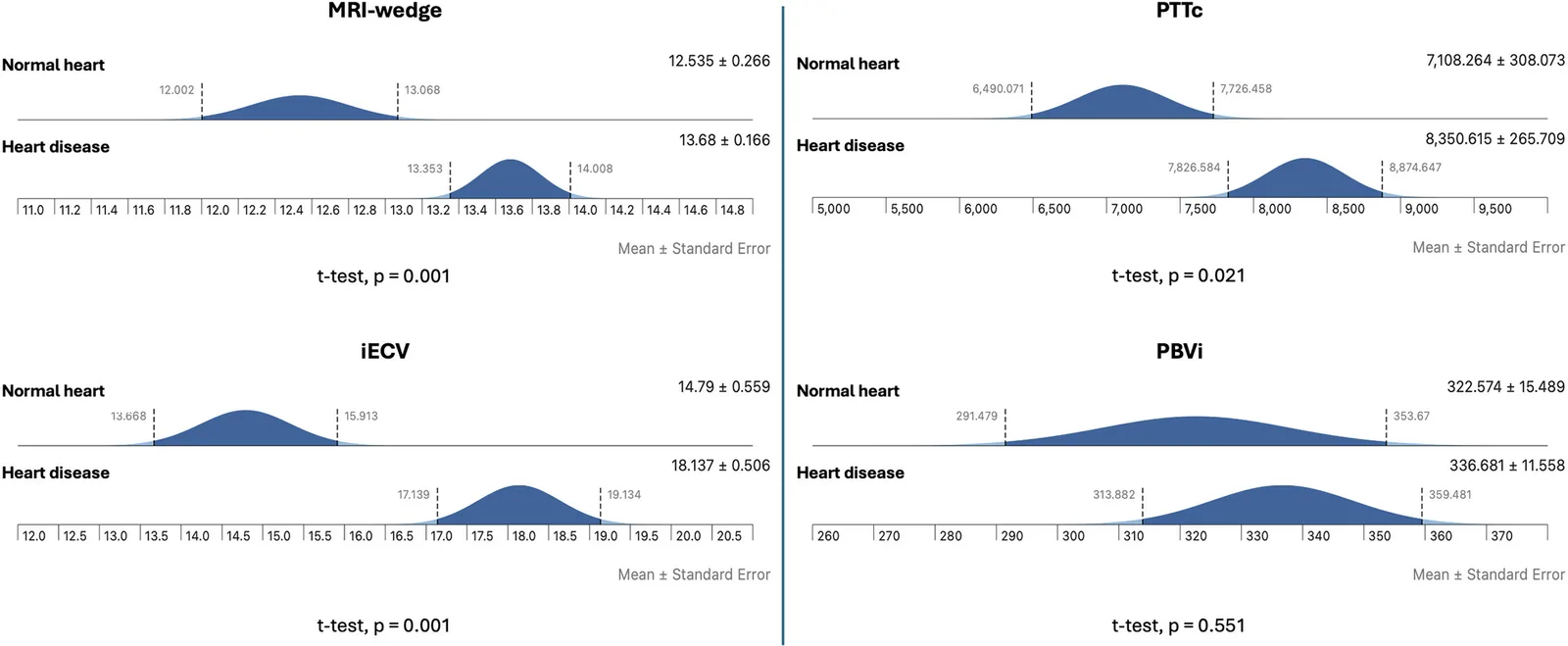
Distribution of MRI-wedge, pulmonary transit metrics and myocardial extracellular volume values stratified by final diagnosis. The figure shows the estimated density distributions of MRI-wedge, PTTc, PBVi, and iECV in individuals with a normal heart compared with those diagnosed with heart disease. Mean ± standard error for each group is reported to the right of each distribution, together with the corresponding p values from group comparisons. MRI-wedge, cardiovascular magnetic resonance-derived wedge pressure; PTTc, pulmonary transit time corrected for heart rate; PBVi, pulmonary blood volume index; iECV, indexed extracellular volume

MRI-derived wedge pressure was higher in patients with cardiac disease compared with those with normal MRI findings (*p* = 0.001). Similarly, iECV showed significantly higher values in the cardiac disease group (*p* = 0.001). PTTc was modestly prolonged in patients with cardiac disease (*p* = 0.021), whereas PBVi did not differ significantly between groups (*p* = 0.551) (Fig. 2).

### MRI-wedge and indices of myocardial fibrosis

**Fig. 3:**
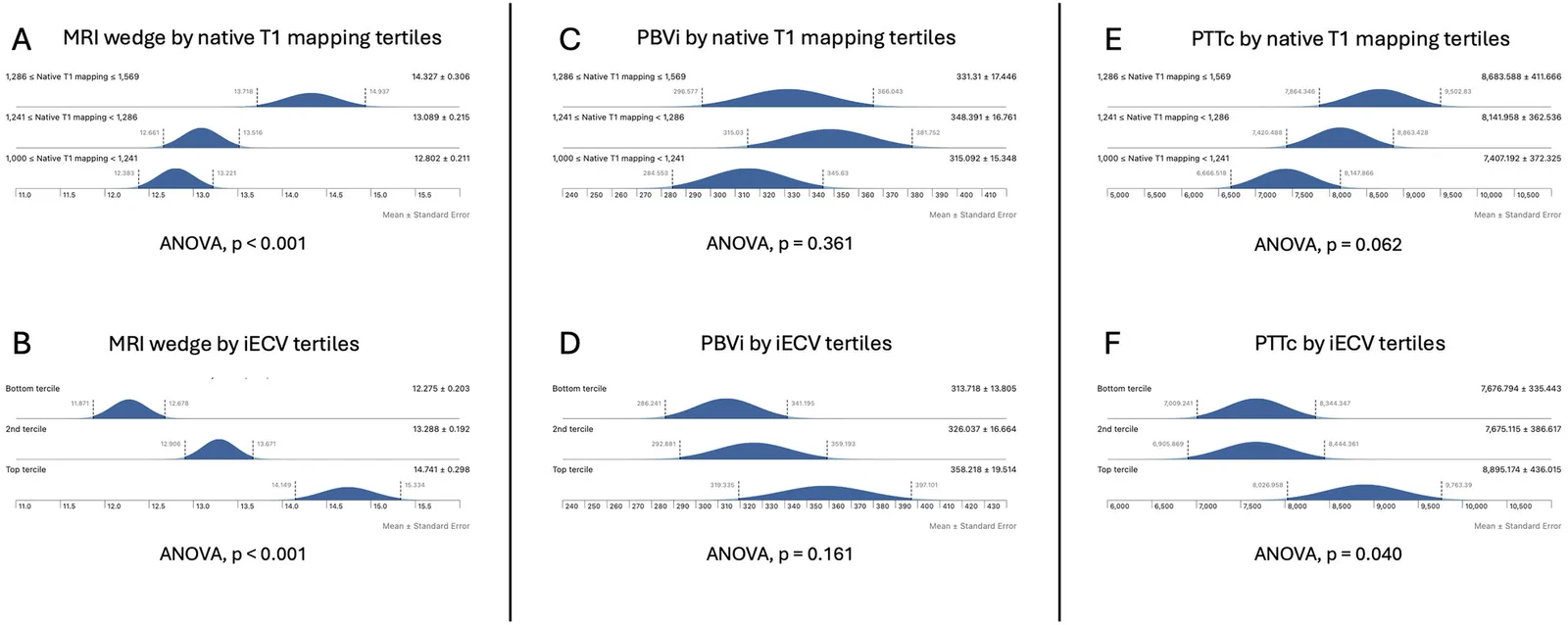
MRI-wedge and pulmonary transit physiology in relation to T1 mapping indices. **A**, **B** Illustrate the distribution of MRI-wedge values across tertiles of myocardial fibrosis markers. **A** MRI-wedge stratified by global native T1 mapping tertiles, showing a statistically significant relationship (ANOVA, *p* < 0.001). **B** MRI-wedge stratified by indexed extracellular volume fraction (iECV) tertiles, also demonstrating a statistically significant relationship (ANOVA, *p* < 0.001). These findings indicate that MRI-wedge is significantly associated with myocardial fibrosis markers, particularly T1 mapping and iECV. **C**, **D** Illustrate the distribution of PBVi values across tertiles of different markers of myocardial fibrosis. **C** PBVi values stratified by global native T1 mapping tertiles showed no significant association (ANOVA, *p* = 0.361). **D** PBVi values stratified by iECV tertiles showed no significant association (ANOVA, *p* = 0.161). **E**, **F** Illustrate the distribution of PTTc values across tertiles of different markers of myocardial fibrosis. **E** PTTc values stratified by global native T1 mapping tertiles showed no significant association (ANOVA, *p* = 0.062). **F** PTTc values stratified by iECV tertiles showed only a weak association (ANOVA, *p* = 0.040). MRI-wedge, cardiovascular magnetic resonance-derived wedge pressure; iECV, indexed extracellular volume; PBVi, pulmonary blood volume index; PTTc, corrected pulmonary transit time

MRI-wedge demonstrated modest but significant correlations with global native T1 (*r* = 0.203, *p* = 0.001) and with iECV (*r* = 0.466, *p* < 0.001), as shown in Supplementary Fig. 1. ANOVA confirmed graded increases in MRI-wedge across tertiles of native T1 mapping (*p* < 0.001) and iECV (*p* < 0.001) (Fig. 3A, B). No significant association was observed between MRI-wedge and myocardial T2 mapping (*p* = 0.06). No significant association was observed between MRI-derived wedge pressure and PALS. Pearson correlation analysis confirmed the absence of a linear relationship (*r* = −0.08, *p* = 0.228), with wide dispersion of values across the full PALS range.

### MRI-wedge and pulmonary transit metrics

Patients with MRI-wedge ≥ 15 mmHg had higher PBVi than those with MRI-wedge < 15 mmHg (413.6 ± 27.4 mL/m² vs 312.4 ± 9.3 mL/m²; *p* < 0.001). PTTc was also greater in the elevated-wedge group (10,026 ± 626 ms vs 7561 ± 210 ms; *p* < 0.001). Across the whole cohort, MRI-wedge demonstrated modest but statistically significant correlations with PBVi (*r* = 0.219; *p* < 0.001) and with PTTc (*r* = 0.231; *p* < 0.001).

### Pulmonary transit metrics and indices of myocardial fibrosis

PTTc and PBVi showed no significant differences across tertiles of global native T1 (PTTc *p* = 0.062; PBVi *p* = 0.361). Across iECV tertiles, PBVi again showed no significant variation (*p* = 0.161), whereas PTTc demonstrated a modest but statistically significant difference (*p* = 0.04) (Fig. 3C–F). Pearson analysis showed a weak association between iECV and PTTc (*r* = 0.164, *p* = 0.011), whereas no significant correlation was found between iECV and PBVi (*r* = 0.114, *p* = 0.08) (Supplementary Fig. 2).

### Clinical outcomes

**Fig. 4:**
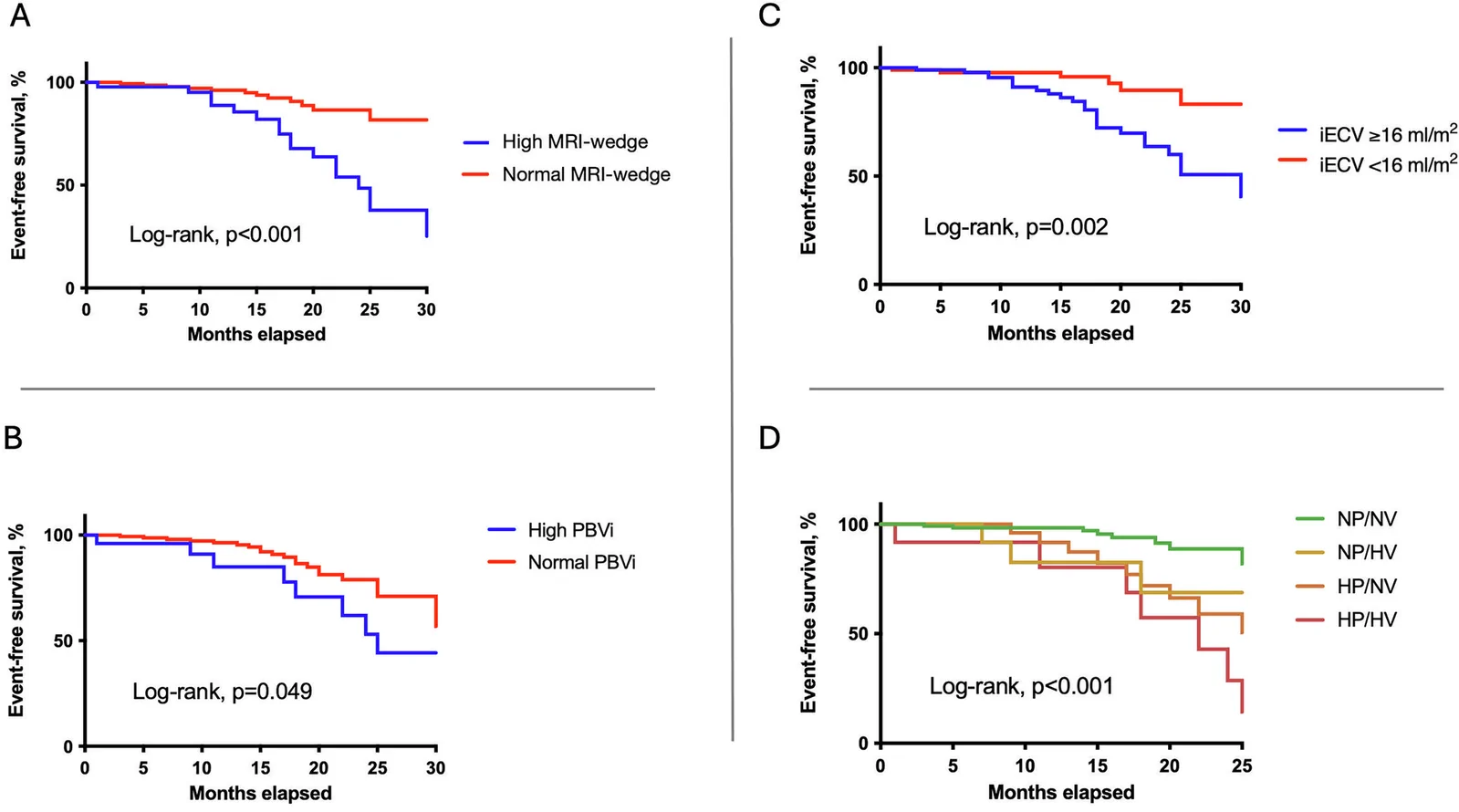
Survival analysis: composite endpoint of cardiovascular death and cardiac hospitalisation. Kaplan–Meier survival analysis comparing event-free survival (composite endpoint: cardiovascular death and cardiac hospitalisation) in patients stratified by MRI-wedge (**A**), PBVi (**B**), and extracellular volume fraction indexed to body surface area (iECV) (**C**). The blue curve represents patients with higher values, while the red curve represents patients with lower values. The log-rank test shows a statistically significant difference in survival between the groups, suggesting that higher values of MRI-wedge (*p* < 0.001), PBVi (*p* = 0.049), and iECVi (*p* = 0.002) are associated with worse clinical outcomes. (**D**) Kaplan–Meier survival analysis illustrating event-free survival in patients stratified by MRI-wedge pressure and PBVi. Patients were divided into four groups: • NP/NV (green): normal pulmonary wedge pressure (NP) and normal PBV (NV). • NP/HV (yellow): normal pulmonary wedge pressure (NP) and high PBV (HV). • HP/NV (orange): high pulmonary wedge pressure (HP) and normal PBV (NV). • HP/HV (red): high pulmonary wedge pressure (HP) and high PBV (HV). A MRI-wedge value ≥ 15 mmHg was used to define the HP subgroup, while a PBVi value ≥ 492 mL/m² was used to define the HV subgroup. The log-rank test indicates a statistically significant difference (*p* < 0.001) in survival among the groups, with the HP/HV subgroup showing the worst prognosis. MRI-wedge, MRI-derived wedge pressure; PBVi, pulmonary blood volume index; iECV, indexed extracellular volume; NP, normal pulmonary wedge pressure; NV, normal pulmonary blood volume; HV, high pulmonary blood volume

Of the 262 individuals included in the imaging analysis, 23 were lost to follow-up, leaving 239 patients (91%) with available outcome data. During a median follow-up of 30 months, 29 patients (12%) reached the composite endpoint. Events included cardiovascular death (*n* = 8), heart-failure hospitalisation (*n* = 10), and cardiac device implantation (*n* = 11). Kaplan–Meier curves demonstrated lower event-free survival in patients with elevated MRI-wedge (*p* < 0.001) (Fig. 4A), expanded iECV (*p* = 0.002) (Fig. 4B), and increased PBVi (*p* = 0.01) (Fig. 4C). Integration of MRI-wedge and PBVi revealed a clear gradient of risk across the four haemodynamic strata. Patients with normal LVFP (NP < 15 mmHg) and normal PBV (NV < 492 mL/m²) had the most favourable event-free survival, whereas outcomes worsened progressively in groups with isolated abnormalities. The highest risk was observed in patients with concomitantly high LVFP (HP) and high PBV (HV), who exhibited a marked decline in event-free survival (log-rank *p* < 0.001) (Fig. 4D). In the multivariable Cox model, both the MRI congestion stage (*p* = 0.009) and iECV (HR 1.072; 95% CI 1.003–1.146; *p* = 0.04) remained independent predictors of adverse events, while left-ventricular ejection fraction did not retain significance (Table 2). The MRI congestion stage demonstrated a significant overall association with outcomes, and each abnormal stage was linked to a higher risk relative to the reference profile. The reported estimates were HR 4.19 (95% CI 1.03–17.03) for isolated volume overload, HR 4.23 (95% CI 1.48–12.04) for isolated pressure overload, and HR 6.69 (95% CI 1.81–24.74) for the combined pressure–volume overload phenotype, indicating a coherent and graded risk trajectory across the four haemodynamic stages.

**Table 2:** Univariate and multivariate Cox regression analysis

Covariates	Univariate			Multivariate		
	HR	95% CI	*p*-value	HR	95% CI	*p*-value
Age, years	1.014	0.991–1.038	0.231			
Female sex	0.558	0.238–1.31	0.180			
LVEF, %	0.969	0.949–0.989	0.003			
LVEDVi, mL/m^2^	1.014	1.006–1.022	< 0.001			
LGE, binary	5.485	0.743–40.507	0.095			
iECV, mL/m^2^	1.067	1.031–1.104	< 0.001	1.072	1.003–1.146	0.040
MRI congestion system			< 0.001			0.009
Stage 1	Ref.	-	-	Ref.	-	-
Stage 2	4.204	1.11–15.926	0.035	4.19	1.03–17.03	0.046
Stage 3	6.615	2.652–16.502	< 0.001	4.23	1.48–12.04	0.007
Stage 4	10.94	3.956–30.524	< 0.001	6.69	1.81–24.74	0.004

Univariate and multivariate Cox proportional-hazards models were used to evaluate predictors of the composite endpoint (cardiovascular death and cardiac hospitalisation). Hazard ratios are presented with 95% confidence intervals. Variables included demographic factors, ventricular size and function, late gadolinium enhancement (LGE), iECV, and the MRI haemodynamic congestion system. In the multivariate model, both iECV and the MRI haemodynamic congestion system remained independent predictors of adverse outcomes

### Profiles of the MRI haemodynamic congestion staging system

A structured comparison of imaging and clinical variables across four MRI haemodynamic stages is presented in Table 3. Patients classified as NP/NV represented the largest subgroup (*n* = 171), followed by HP/NV (*n* = 36), NP/HV (*n* = 18), and HP/HV (*n* = 14). Age did not differ significantly across categories. The proportion of male patients was less represented in the NP/NV subgroup (*p* = 0.004). Measurements of left- and right-ventricular structure and function varied substantially. Indexed left ventricular end-diastolic volume increased progressively from NP/NV to HP/HV (73.6 to 127.4 mL/m²; *p* < 0.001). Left ventricular ejection fraction (LVEF) was highest in NP/NV (59.1%) and lowest in HP/HV (36.4%; *p* < 0.001). LV mass index was greater in HP/NV and HP/HV than in the two normal-pressure groups (*p* < 0.001). Left-atrial volume index also differed significantly (*p* < 0.001), with the highest values observed in the HP/HV subgroup. Indexed right ventricular end-diastolic volume and right ventricular ejection fraction showed stepwise variation across categories (both *p* < 0.001). Right-atrial volume index differed across groups (*p* = 0.002). The prevalence of late gadolinium enhancement (LGE) was similar in all stages (*p* = 0.936). Quantitative tissue markers showed significant between-group differences. Native T1 was modestly higher in HP/NV and HP/HV than in NP/NV and NP/HV (*p* = 0.026). ECV and iECV differed markedly (both *p* < 0.001), with higher values in the elevated-pressure strata. Transit physiology indices showed clear gradients: PTTc ranged from 7.2 s in NP/NV to 14.5 s in HP/HV (p < 0.001), and PBVi ranged from 290 to 661 mL/m² across the same groups (*p* < 0.001). MRI-wedge increased from 12.4 to 17.0 mmHg across stages (*p* < 0.001). The proportion of non-ischaemic cardiomyopathy differed significantly across groups (*p* = 0.04), whereas ischaemic heart disease, normal and other MRI categories did not show significant variation.

**Table 3:** Profiles of MRI haemodynamic congestion stages

MRI haemodynamic congestion stages	Stage 1 Normal profile (NP/NV)	Stage 2 Volume overload (NP/HV)	Stage 3 Pressure overload (HP/NV)	Stage 4 Combined overload (HP/HV)	*p*-value
	*n* = 171	*n* = 18	*n* = 36	*n* = 14	
Age, years	52.0	46.1	55.6	53.7	0.326
Male sex, %	59.6	88.9	80.6	85.7	0.004
BSA, m²	1.86	1.86	1.97	1.87	0.043
LVEDVi, mL/m²	73.6	105.1	90.9	127.4	< 0.001
LVEF, %	59.1	54.3	49.9	36.4	< 0.001
LV mass index, g/m²	64.8	70.7	83.5	76.2	< 0.001
LV MWT, mm	10.4	10.1	12.0	10.5	0.058
LAVi, mL/m²	26.8	26.2	49.4	57.7	< 0.001
RVEDVi, mL/m²	67.4	86.9	73.9	98.1	< 0.001
RVEF, %	61.2	55.8	56.8	49.6	< 0.001
RAVi, mL/m²	45.6	52.6	50.2	61.5	0.002
LGE, %	75	78	80	79	0.936
Native T1, ms	1259	1257	1293	1284	0.026
ECV, %	25.5	24.0	29.1	28.0	< 0.001
iECV, mL/m²	15.8	16.3	23.7	20.2	< 0.001
PTTc, s	7.2	12.4	8.5	14.5	< 0.001
PBVi, mL/m²	290.5	564.0	326.3	661.4	< 0.001
MRI wedge, mmHg	12.4	12.6	16.7	17.0	< 0.001
Adjudicated diagnosis, %					
IHD	33	1	10	5	0.470
NICM	65	13	16	5	0.040
Other	28	1	10	5	0.054
Normal MRI	42	3	4	2	0.291
Event rate, %	4.7	16.7	30.6	50	< 0.001

## Discussion

This study demonstrates that MRI-derived estimates of LVFP, PBV, and myocardial tissue remodelling provide complementary information on cardiac structure, haemodynamics and identify patients at increased risk of adverse events in a broad all-comer population referred to MRI for suspected cardiac pathology. The addition of a combined staging framework-based on MRI-wedge and PBVi— further clarifies how these markers organise along a continuum of congestion biology and identify phenotypes with distinct structural and functional signatures.

Higher MRI-wedge values were closely aligned with features of more advanced structural involvement, including larger atrial and ventricular volumes, greater LV mass, and higher native T1 and iECV, consistent with sustained pressure load and interstitial matrix activation. This pattern was observed across diverse diagnostic groups and was independent of left-ventricular systolic function, supporting the interpretation of MRI-wedge as a structural surrogate that reflects chronic haemodynamic stress rather than transient physiological variation.

By comparison, measures of pulmonary transit physiology showed a more selective pattern: although PBVi and PTTc did not uniformly correlate with tissue markers and showed wider dispersion, abnormalities in these parameters identified patients with more advanced circulatory involvement, suggesting that pulmonary vascular adaptation emerges later, when elevation of LVFP becomes sustained, and central circulatory reserve is exceeded. Consistent with this, individuals with elevated LVFP showed higher PBV and longer transit times, suggesting a measurable redistribution of central blood volume. These findings were consistent with the four-stage classification, in which patients with normal LVFP and normal PBV exhibited the most favourable MRI profile, and those with combined elevation of both indices showed the most pronounced structural and tissue abnormalities.

Clinical follow-up confirmed the relevance of this integrated assessment. Event-free survival declined in patients with elevated MRI-wedge, higher PBVi, expanded iECV, and across the four MRI haemodynamic stages. The combined overload group demonstrated the lowest survival, consistent with its more advanced structural and tissue characteristics. In multivariable analysis, the MRI haemodynamic congestion system and iECV remained independent predictors of the composite endpoint, supporting the complementary clinical value of haemodynamic assessment and quantitative tissue-level characterisation. These findings indicate that a staging approach integrating LVFP and PBV captures clinically meaningful differences in disease expression and that diffuse myocardial remodelling adds incremental prognostic information.

The strong association between MRI-wedge and myocardial remodelling observed in our population is consistent with prior work demonstrating that left-atrial enlargement and ventricular hypertrophy encode the cumulative burden of elevated LVFP, and is in agreement with population-level data showing that demographic and clinical determinants of raised LVFP closely parallel chamber dilatation and chronic structural adaptation [17]. Structural pressure surrogates derived from MRI have been shown to approximate invasively measured PAWP with high fidelity and superior reproducibility compared with Doppler-based indices [13, 14]. Although the initial prognostic study of MRI-wedge was performed in a single-centre cohort and may therefore have been susceptible to referral bias, subsequent studies have provided supportive evidence across HF, population-based, and disease-specific cohorts [18–21]. In a multicentre prospective setting, the model maintained diagnostic discrimination in patients evaluated for suspected HF and reproduced prognostic stratification traditionally achieved through catheterisation [22]. Our findings extend these observations by confirming that MRI-based pressure estimation retains biological and clinical validity even when applied to diverse clinical populations. The relationship between MRI-wedge and diffuse myocardial remodelling reflects a central pathophysiological connection where chronic haemodynamic load promotes extracellular matrix expansion, and the expansion itself drives stiffness, diastolic dysfunction, haemodynamic deterioration and symptoms. This aligns with recent evidence that non-invasive MRI-based estimates of LVFP correlate with circulating and echocardiographic markers of congestion, including pulmonary findings on lung ultrasound in patients with HF and reduced ejection fraction [22]. Prior studies have demonstrated that native T1 and myocardial extracellular matrix increase in proportion to diastolic stiffness and LVFP in HF with preserved ejection fraction, aortic stenosis, hypertrophic cardiomyopathy, and systolic dysfunction [16, 23, 24]. Recent CMR data further extend this concept to the post-infarction setting, showing that extracellular volume (ECV) measured in the remote, non-infarcted myocardium was independently associated with major adverse cardiac events in patients with STEMI [25]. In that cohort, the primary endpoint was a composite of all-cause death, reinfarction, and new congestive HF, supporting the prognostic relevance of extracellular matrix expansion beyond the infarct core itself. Similar associations have been described in cardiac transplant recipients, where diffuse fibrosis correlates with impaired compliance and late allograft dysfunction [26]. The parallel rise of MRI-wedge and iECV in our study reinforces these mechanistic observations, indicating that structural surrogates of pressure load and quantitative markers of interstitial remodelling capture complementary aspects of the same remodelling pathway.

Pulmonary transit metrics showed only a modest association with iECV, suggesting that pulmonary vascular adaptation becomes evident only when interstitial remodelling and sustained elevation of LVFP have progressed. Prior studies support this hierarchical pattern whereby PBV and PTT increase only once left-sided congestion becomes sustained, and pulmonary vascular capacitance is exceeded, signalling a later stage of circulatory compromise [8, 27]. Additional analyses in cardiomyopathies showed that PBV elevation is most pronounced in patients with advanced hypertrophic or dilated phenotypes, reflecting late circulatory consequences rather than early structural change [7, 15]. Additional insight comes from congenital heart disease: patients with repaired lesions frequently exhibit prolonged transit time and increased PBV, closely tracking chamber dilation, impaired ventricular performance, and neurohormonal activation [28]. Although the underlying anatomical substrates differ across studies, the physiological message is consistent—transit-based metrics become abnormal mainly when congestion is long-standing and cardiopulmonary reserve is reduced [29]. Integrated with our data, this supports a framework in which pulmonary transit indices represent downstream markers of circulatory adaptation, complementary to earlier indicators of myocardial remodelling.

The prognostic analysis reinforces the central observation of this study: indices capturing haemodynamic load and myocardial tissue composition each convey meaningful, and independent, information about clinical risk. The MRI haemodynamic congestion system and iECV remained significant predictors of cardiovascular death or cardiac hospitalisation, indicating that both the pressure–volume axis and the tissue-remodelling axis contribute to the pathways through which congestion evolves and clinical deterioration ensues. A graded pattern emerged across the four haemodynamic stages defined by LVFP and PBVi. Patients with normal values for both parameters formed a physiological reference, characterised by normal chamber sizes, preserved biventricular function, and minimal tissue abnormalities. Elevation of PBVi in the absence of raised LVFP identified an early, volume-dominant phenotype, whereas isolated elevation of LVFP was associated with more pronounced structural remodelling and higher iECV. The combined elevation of both indices marked a late and more adverse stage, with substantial chamber dilatation, reduced systolic performance, expanded iECV, prolonged transit, and the highest event rates. This orderly progression suggests that the staging framework captures a biologically coherent sequence spanning early circulatory adaptation to overt haemodynamic compromise.

Overall, these findings outline an integrated, non-invasive MRI framework in which structural pressure surrogates, myocardial tissue characterisation, and transit physiology define a spectrum of haemodynamic congestion. The distinct and additive prognostic contributions of the haemodynamic staging system and iECV underscore the complementary roles of load and interstitial remodelling in the progression of cardiac disease. Although this study was not designed to inform treatment decisions, the proposed MRI haemodynamic staging system may have potential implications for clinical management. Patients with isolated or combined abnormalities could be considered for closer follow-up and a lower threshold for reassessment of volume status or optimisation of decongestive therapy, whereas those with a normal haemodynamic profile may require less intensive surveillance. Validation in larger and disease-specific cohorts, ideally with incorporation of invasive haemodynamic data, and prospective evaluation in management-guided studies will clarify how this approach may refine clinical assessment and inform therapeutic decision-making.

### Strengths and limitations

Several features strengthen the interpretation of our findings. The MRI protocol integrated structural modelling, transit dynamics, and quantitative tissue characterisation within a single examination, enabling a multidimensional assessment of congestion not achievable with conventional imaging approaches. Notably, the haemodynamic metrics evaluated in this study are derived from routinely acquired first-pass perfusion imaging and subsequent post-processing of signal intensity (SI) curves. As such, they can be obtained from standard contrast-enhanced MRI datasets without requiring additional imaging sequences, extra contrast administration, or prolongation of the examination time, thereby preserving the efficiency of a routine clinical MRI protocol. The inclusion of individuals with entirely normal MRI studies provided a true physiological reference, rather than relying solely on disease-based comparisons. In addition, follow-up captured clinically meaningful events that align with the biological mechanisms under investigation.

This study also has limitations. The moderate sample size and the single-centre design may limit generalizability. Patients with atrial fibrillation or frequent ventricular ectopy not responsive to antiarrhythmic therapy were excluded; given that these rhythm disorders are independently associated with higher cardiovascular risk, their exclusion may have led to a conservative estimate of absolute event rates. Although the number of clinical events was sufficient for the primary analysis, it limited detailed exploration of disease-specific subgroups. In addition, although the final multivariable model was deliberately restricted to a parsimonious structure in order to limit dimensionality and reduce the risk of overfitting, the moderate number of outcome events may still constrain the precision of regression estimates, particularly for continuous variables. Accordingly, hazard ratios should be interpreted with appropriate caution, and further confirmation in larger cohorts will be important to refine the stability and external validity of these estimates. Although the study was conducted in a stable outpatient setting, in which variability related to acute congestion or treatment changes is expected to be limited, comprehensive clinical characterisation of HF severity—including functional status, serum biomarkers of congestion and myocardial injury, and background medical therapy—was not systematically captured and therefore was not incorporated into multivariable modelling. Consequently, the incremental prognostic value of these novel variables beyond established clinical indicators cannot be determined within the present cohort and warrants evaluation in future integrated models. Finally, invasive hemodynamic confirmation was not available.

## Conclusions

In this clinical cohort, MRI-derived indices of haemodynamic load and myocardial tissue composition provided complementary and clinically meaningful information. The proposed MRI haemodynamic framework, based on the combined assessment of LVFP and PBV, delineated a coherent spectrum of haemodynamic burden ranging from physiological haemodynamics to advanced structural and circulatory compromise, and was independently associated with the risk of cardiovascular events. Indexed ECV provided additional prognostic insight, reflecting the contribution of diffuse myocardial remodelling to adverse outcomes. Taken together, these findings indicate that a single, non-invasive MRI examination can characterise the principal domains through which congestion develops—structural load, interstitial remodelling, and central circulatory adaptation—and can stratify risk across a broad range of cardiac conditions. Further validation in larger and disease-specific populations will clarify how this integrated framework may inform clinical decision-making and support earlier, physiology-guided intervention.

## Supplementary information

Supplementary information

## Abbreviations

ANOVA: Analysis of variance
ECV: Extracellular volume
HF: Heart failure
iECV: Indexed extracellular volume
LA: Left atrium
LAV: Left atrial volume
LGE: Late gadolinium enhancement
LVEF: Left ventricular ejection fraction
LVFP: Left ventricular filling pressure
LVM: Left ventricular mass
MRI: Magnetic resonance imaging
PALS: Peak left atrial longitudinal strain
PAWP: Pulmonary artery wedge pressure
PBV: Pulmonary blood volume
PBVi: Pulmonary blood volume index
PC: Phase-contrast
PTB: Pulmonary transit beats
PTTc: Corrected pulmonary transit time
RA: Right atrium
RHC: Right heart catheterization
RVSV: Right ventricular stroke volume
