# Supplementary material for: MRI staging of haemodynamic congestion and clinical outcomes
Source: Eur Radiol. 2026 Jun 10;36(10):7898–910. doi: 10.1007/s00330-026-12676-4 (PMC13574865; doi:10.1007/s00330-026-12676-4)
Supplement: Supplementary file 1 [file 330_2026_12676_MOESM1_ESM.pdf]

ELECTRONIC SUPPLEMENTARY MATERIAL

MRI Staging of Haemodynamic Congestion and Clinical Outcomes

Supplementary Figure 1

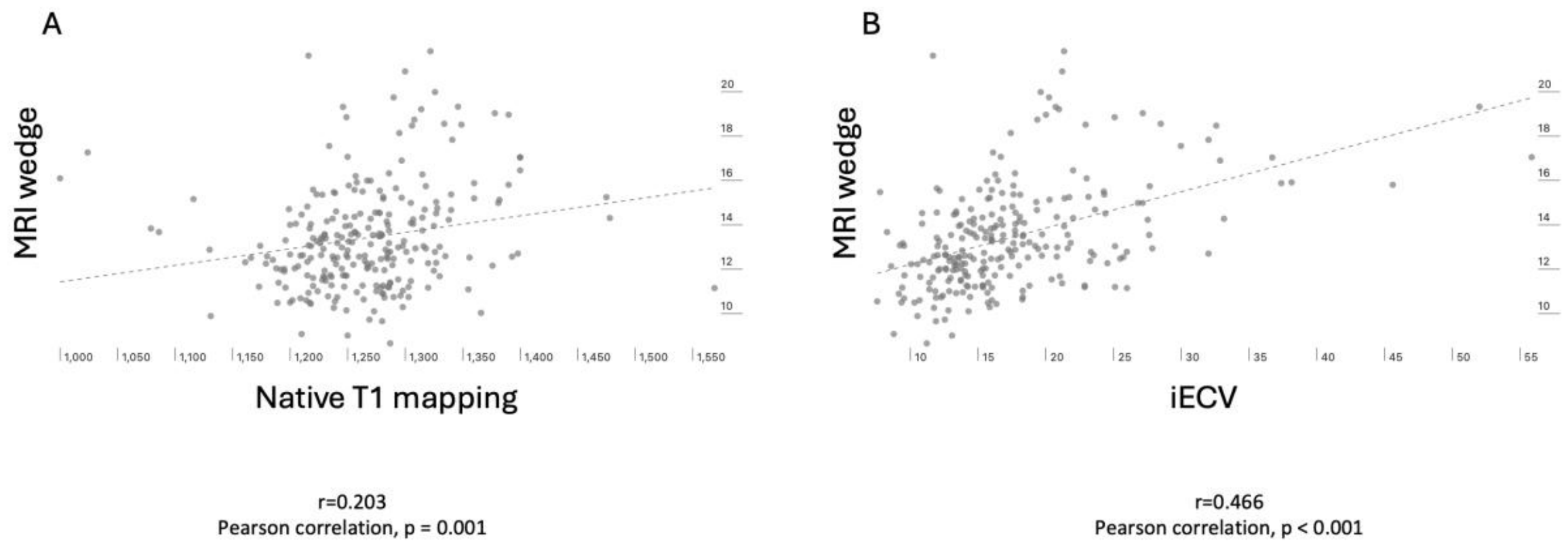

Correlation (Pearson analysis) between MRI wedge and myocardial fibrosis markers (T1 mapping and iECV). Panel A illustrates the relationship between MRI wedge and global native T1 mapping values, showing a statistically significant positive correlation ($r = 0.203$, $p = 0.001$). Panel B demonstrates a significant positive correlation between MRI wedge and iECV ($r = 0.466$, $p < 0.001$). These findings suggest that higher MRI wedge values are associated with increased myocardial fibrosis, supporting its role as a non-invasive marker of hemodynamic congestion and myocardial remodeling. MRI wedge: magnetic resonance imaging-derived wedge pressure; iECV: indexed extracellular volume.

Supplementary Figure 2

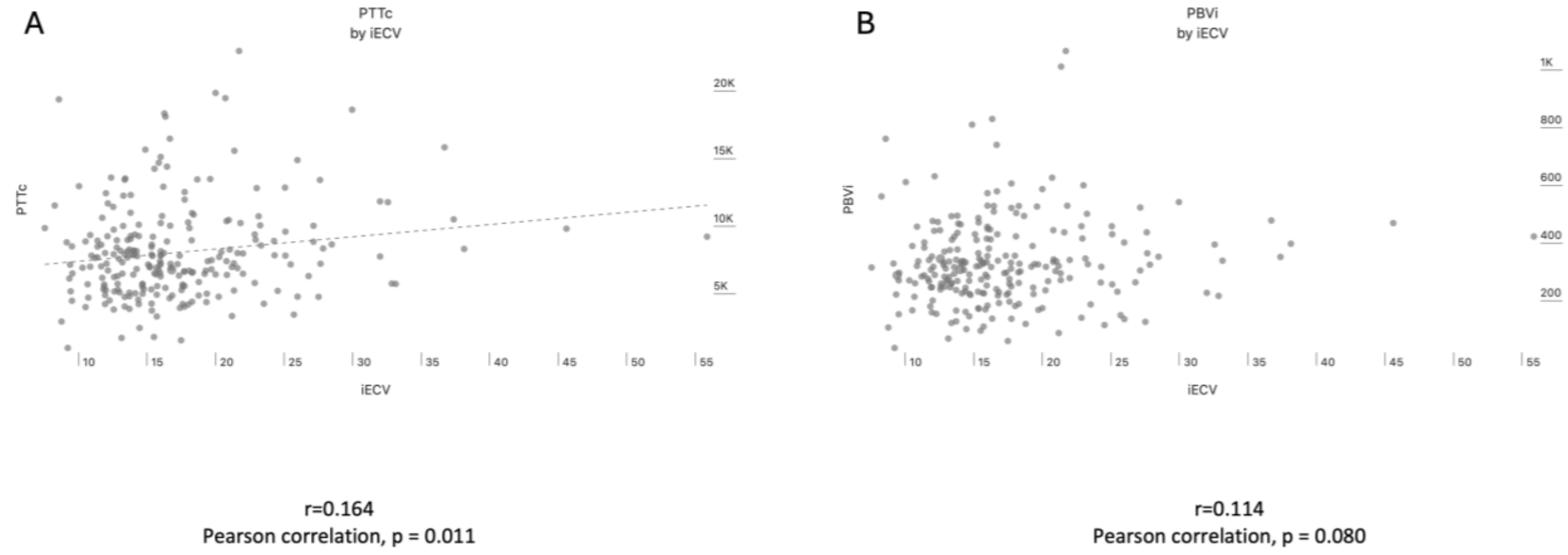

Scatter plots illustrating the correlation between indexed extracellular volume (ECVi) and two magnetic resonance imaging (MRI)-derived metrics: corrected pulmonary transit time (PTTc) (A) and pulmonary blood volume index (PBVi) (B). Pearson correlation analysis demonstrates a weak positive correlation between PTTc and iECV ($r = 0.164$, $p = 0.011$), while PBVi does not show any correlation with iECVi ($r = 0.114$, $p = 0.080$). Dashed lines indicate the linear regression trend. PTTc: corrected pulmonary transit time; PBVi: pulmonary blood volume index; iECV: indexed extracellular volume.
